# Role and new perspectives of transforming growth factor-α (TGF- ***α***) in adenocarcinoma of the gastro-oesophageal junction

**DOI:** 10.1054/bjoc.1999.1013

**Published:** 2000-02

**Authors:** A D'Errico, C Barozzi, M Fiorentino, R Carella, M Di Simone, L Ferruzzi, S Mattioli, W F Grigioni

**Affiliations:** Pathology Division of the ‘F Addarii’ Institute, Department of Oncology, University School of Medicine, Viale Ercolani 4/2, Bologna, 40138, Italy; Department of Surgery, University School of Medicine, Via Massarenti 9, Bologna, 40138, Italy

**Keywords:** TGF-α, adenocarcinoma, gastro-oesophageal junction

## Abstract

The incidence of gastro-oesophageal junction (GEJ) adenocarcinoma is increasing in Western countries and prognosis is poor since metastasis is most often present at diagnosis. We examined samples from 87 resected type II GEJ adenocarcinomas, 30 of these with endoscopic diagnostic biopsy material, to evaluate transforming growth factor alpha (TGF-α) expression and p53 overexpression by immunohistochemistry and in situ hybridization (for TGF-α), in relation to biological and clinical behaviour. TGF-α messenger RNA (mRNA) and protein were detectable in neoplastic cells in 56% and 64% cases respectively. TGF-α mRNA was detected in intra- and peritumoral lymphocytes and those of metastatic lymph nodes. TGF-α protein expression was significantly associated with tumour progression (*P* = 0.025) and lymph node metastasis (*P*< 0.05). The strong TGF-α expression found in neoplastic cells inside blood and lymphatic vessels and in metastatic localizations suggests that TGF-α-positive GEJ adenocarcinomas could have a more aggressive biological phenotype. The expression of TGF-α mRNA and protein in both inflammatory and neoplastic cells indicates that TGF-α is directly synthesized by both cell compartments. Finally, since TGF-α expression was associated with lymph node metastasis, its detection in preoperative perendoscopic biopsies might identify patients with more aggressive tumours who may need additional therapy, including neo-adjuvant treatment. © 2000 Cancer Research Campaign


British Journal of Cancer (2000) 82(4), 865–870
© 2000 Cancer Research Campaign
DOI: 10.1054/ bjoc.1999.1013, available online at http://www.idealibrary.com on IDEAL®

### Role and new perspectives of transforming growth factor-α (TGF-α) in adenocarcinoma of the gastro-oesophageal junction

**A D'Errico[1], C Barozzi[1], M Fiorentino[1], R Carella[1], M Di Simone[2], L Ferruzzi[2], S Mattioli[2] and WF Grigioni[1]**

[1]Pathology Division of the 'F Addarii' Institute, Department of Oncology, University School of Medicine, Viale Ercolani 4/2, 40138 Bologna, Italy; [2]Department of Surgery, University School of Medicine, Via Massarenti 9, 40138 Bologna, Italy

**Summary** The incidence of gastro-oesophageal junction (GEJ) adenocarcinoma is increasing in Western countries and prognosis is poor since metastasis is most often present at diagnosis. We examined samples from 87 resected type II GEJ adenocarcinomas, 30 of these with endoscopic diagnostic biopsy material, to evaluate transforming growth factor alpha (TGF-α) expression and p53 overexpression by immunohistochemistry and in situ hybridization (for TGF-α), in relation to biological and clinical behaviour. TGF-α messenger RNA (mRNA) and protein were detectable in neoplastic cells in 56% and 64% cases respectively. TGF-α mRNA was detected in intra- and peritumoral lymphocytes and those of metastatic lymph nodes. TGF-α protein expression was significantly associated with tumour progression ($P = 0.025$) and lymph node metastasis ($P < 0.05$). The strong TGF-α expression found in neoplastic cells inside blood and lymphatic vessels and in metastatic localizations suggests that TGF-α-positive GEJ adenocarcinomas could have a more aggressive biological phenotype. The expression of TGF-α mRNA and protein in both inflammatory and neoplastic cells indicates that TGF-α is directly synthesized by both cell compartments. Finally, since TGF-α expression was associated with lymph node metastasis, its detection in preoperative perendoscopic biopsies might identify patients with more aggressive tumours who may need additional therapy, including neo-adjuvant treatment. © 2000 Cancer Research Campaign

**Keywords:** TGF-α; adenocarcinoma; gastro-oesophageal junction

The incidence of gastro-oesophageal junction (GEJ) adenocarcinoma has markedly increased over the last 20–30 years (Blot et al, 1991; Powell and McConkey, 1992; Pera et al, 1993). This contrasts with the decreased incidence of carcinoma of the distal stomach both in Western countries and Japan (Sons and Borchard, 1986; Paterson et al, 1987). Despite improvements in early diagnosis and therapy, the prognosis of GEJ adenocarcinoma remains poor due to the fact that metastasis is already present in most patients at diagnosis (Wang et al, 1993; Jakl et al, 1995). Multimodality treatments have not yet exerted a significant impact on long-term survival, which remains at about 10% at 5 years (MacFarlane et al, 1988; Wolfe et al, 1988; Ohno et al, 1995).

Until recently, most researchers thought of all GEJ adenocarcinoma as an anatomical variety of either distal-oesophageal or gastric adenocarcinoma (Webb and Busuttil, 1978; Bosh et al, 1979; Wang et al, 1986; Stemmermann et al, 1994). Siewert and Stein (1996) have underlined the clinical importance of distinguishing true carcinoma of the cardia arising from epithelium of the GEJ, when the tumour centre is located within 1 cm oral or 2 cm aboral of the anatomical GEJ (type II in their classification), from those originating either in the distal oesophagus (i.e. from Barrett's oesophagus) (type I) or from subcardial gastric mucosa (type III).

Many studies have evidenced the importance of proliferative rate, p53 mutation (evidenced by its overexpression) in the progression of adenocarcinomas of the stomach and distal oesophagus (Casson et al, 1991; Imazeki et al, 1992; Wang et al, 1994): in particular, p53 mutation or protein aberrancy has been correlated with tumour development, invasiveness and poor prognosis (Martin et al, 1992; Wang et al, 1994; Moskaluk et al, 1996; Wu et al, 1998). Transforming growth factor α (TGF-α) has been shown to be an important proliferation activator in various types of epithelial tissue (Malden et al, 1989; Derynk, 1988). Normal gastric mucosa has been shown to express significantly lower levels of TGF-α protein with respect to mucosa adjacent to gastric carcinomas, metaplastic intestinalized mucosa and gastric carcinomas, particularly of the intestinal type (Nasim et al, 1992; Filipe et al, 1995). Advanced gastric carcinomas expressing high levels of both TGF-α protein and epidermal growth factor receptor show a worse prognosis (Yonemura et al, 1992). Furthermore, in patients with oesophageal squamous carcinoma, TGF-α expression has been found to correlate with poor prognosis (Iihara et al, 1993). Sauter et al (1995) found that persistence of (or a rise in) TGF-α protein in adenocarcinomas of the distal oesophagus after treatment with high-dose radiation therapy and chemotherapy correlated with worse prognosis. In contrast, in preoperative colorectal carcinoma biopsies, Younes et al (1996) found that high expression of TGF-α protein is a good prognostic indication. On the other hand, De Jong et al (1998) have reported that TGF-α expression in resected liver metastases from colorectal adenocarcinoma is associated with unfavourable prognosis. These discrepancies prompted us to examine the histological material from 87 type II GEJ adenocarcinomas and their metastatic lymph nodes to investigate how TGF-α mRNA (and its protein) and p53 overexpression correlate with the biological and clinical behaviour of this tumour type.

*Received 28 April 1999*
*Revised 6 October 1999*
*Accepted 6 October 1999*

*Correspondence to*: WF Grigioni

### PATIENTS AND METHODS

### Patients and histological samples

Surgical specimens were studied of GEJ adenocarcinoma, type II according to Siewert and Stein's classification (1996) from 87 patients (20 females, 67 males, mean age 62 years) who underwent total gastrectomy, distal oesophagectomy and extended lymphadenectomy at the Surgical Department of our institution between 1972 and 1996 and had a minimum survival of 2 months (max. 166 months, mean 33 months). In all cases, a radical resection was macroscopically achieved, as evaluated on intraoperative frozen sections. None of the patients had a significant history of gastro-oesophageal reflux. In 30 cases, the preoperative biopsy was performed in our institution, and diagnostic material was available for study by immunohistochemistry and in situ hybridization. Gastric, cardiac and oesophageal samples were taken from each specimen. Foci of intestinal metaplasia were never observed in the oesophageal mucosa. The point of maximum infiltration was sampled in each case. Multiple samples were always taken of the apparently unaffected surrounding gastric and oesophageal tissues. In the absence of a separate staging classification for GEJ adenocarcinoma, American Joint Cancer Committee (AJCC) guidelines for gastric cancer were employed. Accordingly, one patient was classified as stage pT1, 10 were pT2 and 76 were pT3–4. In 28 patients there was no metastatic spread to the lymph nodes, while 35 were N1 and 24 were N2, of whom two were M1 (Table 1). The neoplasms were well to moderately differentiated (G1–2) in 22 patients, poorly differentiated (G3) in 44 patients and undifferentiated (G4) in 21 patients (AJCC criteria). Tissues were fixed in formalin for a maximum of 24 h at 4°C. Fixed material was then processed through absolute ethanol and Histo-clear (histological clearing agent, Atlanta, GA, USA), and paraffin embedded at 58°C in Paraplast Plus (Sherwood Medical, Athy, Ireland) for a maximum of 2 h. Serial 4-μm thick sections were cut from each block, collected on silane precoated slides and allowed to dry overnight at 37°C prior to use. One section of each sample was stained with H&E.

### Immunohistochemistry procedures

Sections were dewaxed and rehydrated through alcohol series up to distilled water. The endogenous peroxidase activity was suppressed by a 0.3% solution of hydrogen peroxide ($H_2O_2$) in methanol for 20 min. After two washes in Tris buffer saline (TBS), sections to be incubated with the anti-human TGF-α monoclonal antibody (mAb) (clone 213–4.4, Calbiochem, Cambridge, MA, USA) diluted 1:100 were processed in a microwave oven in EDTA 1 mM, pH 8 for two cycles of 5 min each. Sections to be incubated with mAb anti-p53 (clone DO-7 Dako, Glostrup, Denmark) diluted 1:80 and polyclonal antibody (pAb) anti-Ki-67 antigen (Dako) diluted 1:50 were processed in a microwave oven in citrate buffer (10 mM, pH 6) for 4 cycles of 5 min. All sections were incubated overnight at 4°C in a humidified chamber. After 3 rinses in TBS, the tissue samples were incubated with the mAb or pAb Envision Plus HRP system (Dako) for 30 min. Immunological reactions were developed in 3,3′-diaminobenzidine (Sigma, St Louis, MO, USA) in TBS (0.6 mg $ml^{-1}$), containing 0.02% $H_2O_2$ for 5 min in the dark. The slides were then counterstained with Mayer's haematoxylin for 5 s, dehydrated and mounted in Eukitt (O Kindler, Freiburg, Germany). Negative controls were performed by substituting the specific primary antibody with non-immune serum.

### Preparation of cRNA probes

The cDNA corresponding to human TGF-α, consisting of 930 bp of the open reading frame of the gene, was sub-cloned in a pGem1 plasmid (2900 bp, Promega, Madison, WI, USA). The plasmid was linearized either with *Eco*RI (Pharmacia Biotech, Uppsala, Sweden) or *Hin*dIII (Gibco-BRL, Milan, Italy) and 1 μg was then used as a template for in vitro synthesis of the sense and antisense probes respectively. Digoxigenin-labelled riboprobes were generated with either SP6 or T7 RNA polymerase (Roche Diagnostics, Milano, Italia), for 2 h at 37°C in 1 × transcription buffer, 35 U of RNAase inhibitor, 1 mM each of ATP, CTP and GTP, as well as 1 mM of a mixture of cold 10 mM UTP and digoxigenin-UTP (6.5 and 3.5 mM respectively) (Roche). For each probe, the length was reduced to a mean size of 50–100 nucleotides by means of alkaline hydrolysis.

### In situ hybridization

Formalin-fixed, paraffin-embedded sections were dewaxed, rehydrated and washed in phosphate-buffered saline (PBS). Sections were digested with proteinase K (2.5 mg $ml^{-1}$) (Sigma, St Louis, MO, USA) in 50 mM Tris–HCl, 5 mM EDTA (pH 7.2) for 30 min at 37°C, fixed in 4% paraformaldehyde in PBS for 5 min at room temperature, and then washed in PBS. Prior to hybridization, sections were dehydrated and air dried. Hybridization was performed at 42–43°C overnight by applying 80–100 ng of digoxigenin-labelled probe in 25 μl of hybridization buffer (50% deionized formamide, 2 × SSC (saline-sodium citrate), 10% dextran sulphate, 1% w/v sodium dodecyl sulphate [SDS]) per section under a glass coverslip. Coverslips were then gently floated off in 5 × SSC. The highest stringency of post-hybridization washes was 60°C in 50% deionized formamide-2 × SSC for 20 min. Sections were rinsed again in 2 × SSC, 0.5 × SSC, 0.1 × SSC, for 15 min each at 37°C, and then equilibrated in Buffer 1 (100 mM Tris–HCl, 150 mM sodium chloride (NaCl) pH 7.5). Anti-digoxigenin pAb antibody diluted 1:500 in Buffer 1 was applied overnight at 4°C. Detection was accomplished with nitro-blue tetrazolium (340 mg $ml^{-1}$)/5-bromo-4-chloro-3-indolyl-phosphate (170 mg $ml^{-1}$) (NBT/BCIP) for 6–8 h (Roche) in buffer 2 (100 mM Tris–HCl, 50 mM magnesium chloride ($MgCl_2$, pH 9.5) in the presence of 1 mM levamisole (Sigma). Each section was counterstained in methyl green, dried at 42°C and glycerol mounted. Controls for the specificity of in situ hybridization results were performed by: (1) pretreatment of tissue sections with RNAase (100 mg $ml^{-1}$) for 1 h at 37°C, before hybridization; (2) hybridization with the TGF-α sense probe; (3) hybridization with the buffer alone, without the probe.

### Statistical analysis

We analysed the clinical-pathological parameters (age, sex and pathological stage) as well as the immunohistochemistry and in situ hybridization results of the investigated markers (proliferative rate, p53, TGF-α protein and transcripts), relating them to the clinical outcome of the patients studied. Data analysis was performed by the SPSS statistical package using Cox regression to study the survival model.

*© 2000 Cancer Research Campaign*

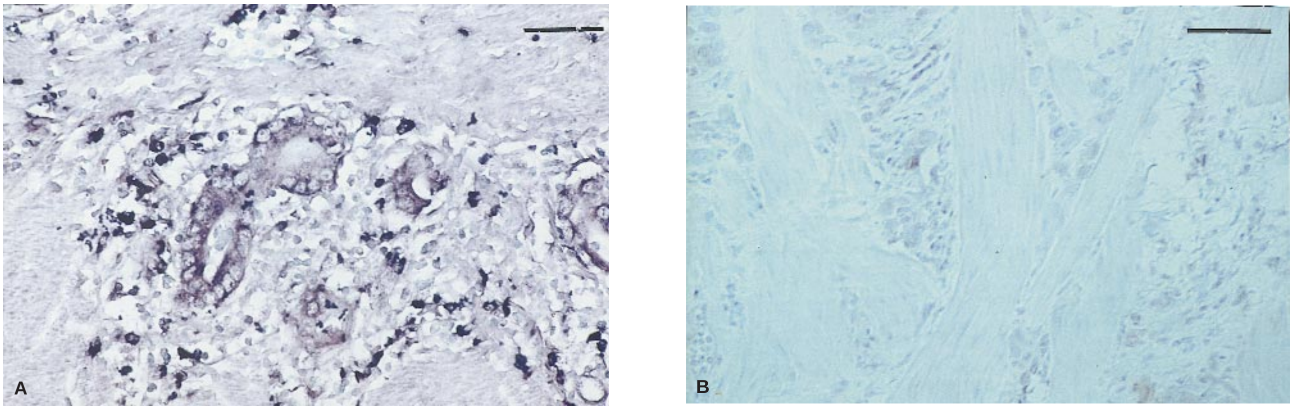

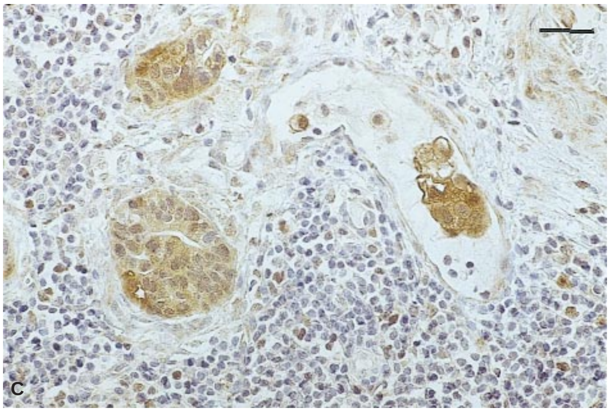

**Figure 1** (**A**) Neoplastic glands and surrounding lymphocytes alongside the invasive front of a GEJ adenocarcinoma show high levels of TGF-α mRNA by in situ hybridization (NBT/AP staining). (**B**) Negative control on the same case using the TGF-α sense probe (NBT/AP staining); (**C**) Neoplastic cells in lymphatic and blood vessels of an infiltrating GEJ adenocarcinoma reveal strong immunostaining using the anti TGF-α mAb (DAB staining)

### RESULTS

Presence of TGF-α transcripts was evidenced by in situ hybridization in the basal layer of the oesophageal epithelium and in basal cells of the gastric glands (both known to be in continuous renewal because of normal turnover). TGF-α messenger RNA (mRNA) was also detected in intra- and peritumoral lymphocytes and those of metastatic lymph nodes. Only scattered positive lymphocytes were detected in normal mucosa and in non-metastatic lymph nodes. High amounts of the transcripts were observed in 49 (56%) cases of adenocarcinoma. In particular, TGF-α mRNA was detected in the majority of neoplastic cells arranged singly or in strings along the line of tumour invasion (Figure 1 A,B) and in the lymphatic or blood vessels. The metastatic lymph-node cells also expressed TGF-α transcripts. TGF-α protein expression was intracytoplasmic and diffuse in the neoplastic cells of 56 (64%) cases (Figure 1C). Co-localization of TGF-α mRNA and protein was nearly always found both in neoplastic cells and in lymphocytes. In seven cases TGF-α protein was present in the absence of its mRNA even in the basal layer of the oesophageal mucosa, almost certainly due to the inevitable degradation of TGF-α mRNA in the oldest archival materials.

Overexpression of p53 protein (Figure 2) was strictly confined to the nuclei of the majority of neoplastic cells of 61/87 (70%) cases (Table 2). Particularly, marked staining for p53 was detected in poorly or undifferentiated carcinomas and in ones with an infiltrating pattern along the oesophageal and gastric walls or with extensive invasion of the submucosa. Furthermore, neoplastic elements in metastatic lymph nodes also showed clear p53 immunoreactivity.

The proliferative index of the neoplasms, as evaluated with the Ki-67 pAb, was scored < 10% in 30 (34%) cases and > 10% in 57 (66%) cases. A higher index was observed in poorly differentiated tumours and in advanced tumour stage (Table 2). Ki-67 positivity was also observed in the basal layer of the squamous epithelium of the oesophagus and in intra- and peritumoral lymphocytes.

In the 30 preoperative biopsies under study, immunohistochemical analysis of p53 and TGF-α and in situ hybridization of TGF-α mRNA showed similar sensitivity and specificity to the surgical

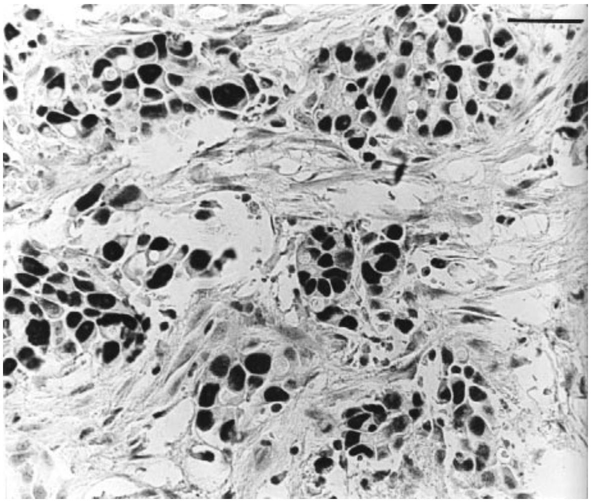

**Figure 2** Immunohistochemical, nuclear localization of p53 protein in a moderately differentiated GEJ adenocarcinoma (DAB staining)

**Table 1** Pathological stage of 87 patients with GEJ adenocarcinoma

| | N0 | N1 | N2 | Total cases |
|---|---|---|---|---|
| pT1 | 1 | 0 | 0 | 1 |
| PT2 | 6 | 3 | 1 | 10 |
| PT3–4 | 21 | 32 | 23 | 76 |

© 2000 Cancer Research Campaign

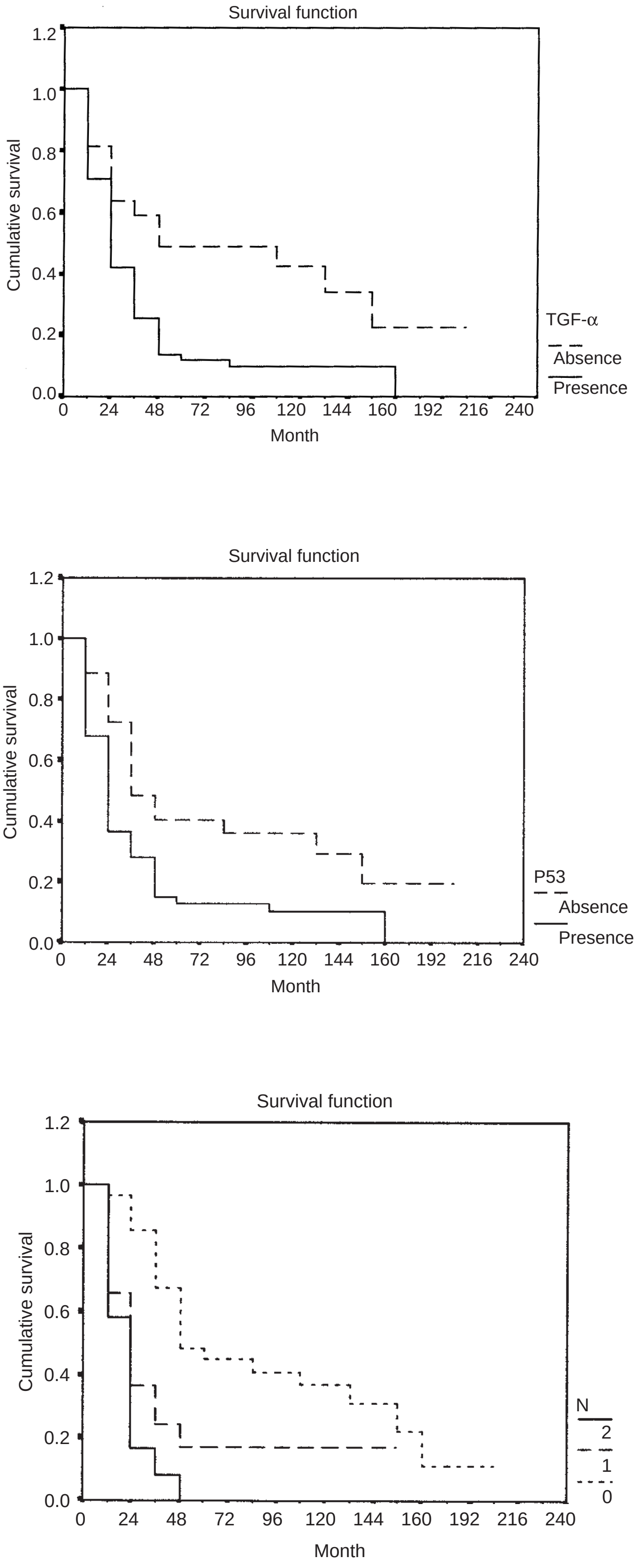

**Figure 3** Cumulative survival for patients with GEJ adenocarcinoma according to TGF-α protein expression (**A**), p53 nuclear accumulation (**B**) and presence of lymph-node metastases (**C**). Clinical outcome is markedly influenced by TGF-α expression ($P$ = 0.0235), p53 accumulation ($P$ = 0.0392) and N stage ($P$ = 0.0025)

**Table 2** Biological findings of 87 patients with GEJ adenocarcinoma

| | No. of cases | TGF-α | TGF-α mRNA | p53 | Ki67>10% | Ki67<10% |
|---|---|---|---|---|---|---|
| pT1 | 1 | 0 | 0 | 0 | 1 | 0 |
| PT2 | 10 | 5 (50%) | 3 (30%) | 5 (50%) | 6 (60%) | 4 (40%) |
| PT3–4 | 76 | 51 (67%) | 46 (52%) | 56 (74%) | 50 (66%) | 26 (34%) |
| Total | 87 | 56 (64%) | 49 (56%) | 61 (70%) | 57 (66%) | 30 (34%) |

samples. In particular, 22/30 preoperative biopsies showed diffuse positivity both for TGF-α protein and mRNA, while the remaining eight cases were negative. This suggests that TGF-α protein and mRNA can both be confidently studied on preoperative endoscopic biopsy specimens.

### Statistical analysis

Cox step-wise regression analysis of the variables (TNM, p53, Ki67 and TGF-α mRNA and protein) showed a significant relationship with increased mortality for p53 ($P$ = 0.030) and N+ ($P$ = 0.0025), while TGF-α protein just missed significance ($P$ = 0.095) due to the masking effect of the variable N+; in effect, logistic regression demonstrated a significant relationship between TGF-α protein and N+ ($P$ = 0.05). When N+ was excluded from the Cox regression, the significant variables were p53 ($P$ = 0.039) and TGF-α protein ($P$ = 0.024); TGF-α mRNA expression failed to reach significance only because of the seven cases that appeared negative due to degradation. Logistic regression also showed that TGF-α protein was significantly associated with tumour progression (i.e. TNM) ($P$ = 0.025). Clinical outcome was not influenced by sex, age or histological grade.

### DISCUSSION

The present study suggests that detection of TGF-α expression is of clinical relevance in type II GEJ adenocarcinomas, as defined by Siewert and Stein (1996), and also sheds new light on the biological action of this growth factor.

On clinical grounds, current issues regarding the treatment of type II GEJ adenocarcinoma include the role of surgery, selection of candidates and the aims of the different operative procedures employed (Sharpe, 1996). Reports from China and Japan have revealed good long-term survival only in patients surgically resected for stage I. Unfortunately, in the USA and Western Europe, even when early diagnosis is made patients are usually already in stage II or III and the modalities of treatment are still undefined.

Activation or suppression of oncogenes and anti-oncogenes has consistently been found to correlate with tumour progression and biological aggressiveness. Expression of their protein products as evaluated on endoscopic biopsy sections can provide useful indications for therapy. In particular, p53 overexpression is known to be a reliable marker of poor prognosis in oesophageal adenocarcinomas (Casson et al, 1995; Patel et al, 1997). As expected, in our series, p53 overexpression correlated negatively with survival ($P$ = 0.030). Its persistence during neoplastic progression and its prognostic significance are thought to be related to a situation of genetic instability associated with the activation of other oncogenes or growth factors (Hartwell, 1992; Wu et al, 1998), including TGF-α.

*© 2000 Cancer Research Campaign*

We found a negative relationship between TGF-α protein and survival ($P$ = 0.024) when the masking effect of N was excluded, suggesting that TGF-α detection could be of prognostic value in type II GEJ adenocarcinomas. Our series confirms the precedence of tumour stage and lymph-node metastasis as overall prognostic indicators, as in most malignancies (Sharpe and Moghissi, 1996; Steup et al, 1996; Kajiyama et al, 1997). However, in Western countries GEJ adenocarcinomas are generally diagnosed in a relatively late stage due to the lack of early signs or symptoms and the tumour's aggressive biological behaviour. Furthermore, clinical staging does not always correspond to the pathological stage of the process, which can be evaluated only after surgery. Thus, biological markers that can provide therapeutic indications at the time of endoscopic biopsy may be especially welcome for GEJ adenocarcinoma patients.

Immunohistochemical assessment of p53 protein overexpression seems to be the most important current biological prognostic marker in GEJ adenocarcinomas. However, evaluation of p53 tumour-suppressor gene mutations (rather than indirect immunohistochemical assessment of the protein accumulation) would require DNA sequencing after single-strand conformational polymorphism polymerase chain reaction (SSCP-PCR). In certain cases, this distinction has been found to be important (Coggi et al, 1997). Thus, we think that TGF-α expression may provide further information on the biological behaviour of type II GEJ adenocarcinomas that could be useful for clinical management.

In our study, expression of TGF-α mRNA and its protein product were detectable in the neoplastic cells in 56% and 64% of cases, respectively, as well as within the blood and lymphatic vessels and in the metastatic lymph nodes, suggesting that neoplastic cells which express TGF-α have a more aggressive biological phenotype. This could be due either to: (1) a direct chemotactic effect of TGF-α; (2) interaction of TGF-α with other growth factors or adhesion molecules; (3) a possible role of TGF-α in the induction of angiogenesis within the tumour, as previously suggested (Schreiber et al, 1986; Cai et al, 1997); (4) concomitant autocrine controlled proliferation and protection from apoptosis (Foster et al, 1999). Interestingly, in adenocarcinomas of the distal oesophagus, Sauter et al (1995) have shown that persistent or increased expression of TGF-α after neo-adjuvant therapy is related to poor prognosis. Taken together, these observations make us think that in type II GEJ adenocarcinomas TGF-α detection at endoscopic biopsies taken before and after neoadjuvant treatment might provide important therapeutic indications, independently of TNM.

From the biological standpoint, we were interested to find that the lymphocytes in and around the tumour and in the metastatic lymph nodes also strongly expressed TGF-α mRNA and protein. Recent studies have shown that in several non-neoplastic intestinal pathologies, the inflammatory component is capable of activating growth factors that stimulate epithelial proliferation, thereby inducing crypts hyperplasia (Bajaj-Elliott et al, 1998). Other studies have focused on the role of the inflammatory component and its prognostic significance in different malignant tumours, along with the mechanism by which some cytokines can either encourage or inhibit tumour growth (Ropponen et al, 1997; Naito et al, 1998). Our observation of co-localization of TGF-α mRNA and protein in both inflammatory and neoplastic cells indicates that TGF-α is directly synthesized by both cell compartments. Moreover, the strong TGF-α expression in neoplastic cells within blood vessels at the edge of the tumours seems to support the hypothesis that production of this protein is associated with tumour progression.

In conclusion, we would like to propose detection of TGF-α protein expression as a new marker to help predict the biological behaviour of type II GEJ adenocarcinomas at the time of endoscopic biopsy and decide on their clinical management. On biological grounds, we think that TGF-α may exert its biological effect by a dual autocrine/paracrine mechanism. This invites further investigation on the role of TGF-α in other epithelial malignancies.

### ACKNOWLEDGEMENT

We are grateful to Dr Roberto Bolzani for statistical analysis. Mr Robin M T Cooke helped revise the manuscript

### REFERENCES

American Joint Committee on Cancer (1997) Cancer Staging Manual, pp. 71–76, 5th edition. Lippincott-Raven: Philadelphia

Bajaj-Elliott M, Poulsom R, Pender SL, Wathen NC and Macdonald TT (1998) Interactions between stromal cell-derived keratinocyte growth factor and epithelial transforming growth factor in immune-mediated crypt cell hyperplasia. *J Clin Invest* **102:** 1473–1480

Blot WJ, Devesa SS, Kneller RW and Fraumeni JF (1991) Rising incidence of adenocarcinoma of the oesophagus and gastric cardia. *JAMA* **265:** 1287–1289

Bosh A, Frias Z and Caldwell WL (1979) Adenocarcinoma of the esophagus. *Cancer* **43:** 1557–1560

Cai Y-C, Barnard G, Hiestand L, Woda B, Colby J and Banner B (1997) Florid angiogenesis in mucosa surrounding an ileal carcinoid tumor expressing transforming growth factor α. *Am J Surg Pathol* **21:** 1373–1377

Casson AG, Kerkvliet N and Omalley F (1995) Prognostic value of p53 protein in esophageal adenocarcinoma. *J Surg Oncol* **60:** 5–11

Casson AG, Mukopadhay T, Cleary KR, Ro JY, Levin B and Roth JA (1991) p53 mutations in Barrett's epithelium and esophageal cancer. *Cancer Res* **51:** 4495–4499

Coggi G, Bosari S, Roncalli M, Graziani D, Bossi P, Viale R, Buffa R, Ferrero S, Piazza M, Blaudamura S, Segalin A, Bonavina L and Peraculia A (1997) p53 protein accumulation and p53 gene mutation in esophageal carcinoma. A molecular and immunohistochemical study with clinicopathological correlations. *Cancer* **79:** 425–432

De Jong KP, Stellema R, Karrenbeld A, Lousdtaal J, Gouw AS, Sluiter WJ, Peeters PMJ, Slooff MJH and Devries EGE (1998) Clinical relevance of transforming growth factor α, epidermal growth factor receptor, p53 and Ki67 in colorectal liver metastases and corresponding primary tumours. *Hepatology* **28:** (4): 971–979

Derynk R (1988) Transforming growth factor α. *Cell* **54:** 593–595

Filipe MI, Osborn M, Linehan J, Sanidas E, Brito MJ and Jankowski J (1995) Expression of transforming growth factor alpha, epidermal growth factor receptor and epidermal growth factor in precursor lesions to gastric carcinoma. *Br J Cancer* **71:** 30–36

Foster JM, Willis J, Venkaeswarlu S, Lin Y, Ferguson K, Zboroskwa E, Brattan MG and Willson JKV. (1999) Ectopic TGF-α expression in non-tumorigenic colon cancer cells results in *in vivo* progression by induction of angiogenesis and proliferation, and inhibition of apoptosis. *Proceedings of the American Association for Cancer Research* Vol. 40 (Abstract 1166)

Hartwell L (1992) Defects in a cell-cycle checkpoint may be responsible for the genomic instability of cancer cells. *Cell* **7:** 543–546

Husemann B (1989) Cardia carcinoma considered as a distinct clinical entity. *Br J Surg* **76:** 136–139

Iihara K, Shiozaki H, Tahara H, Kobayashi K, Inoue M, Tamura S, Miyata M, Oka H, Doki Y and Mori T (1993) Prognostic significance of transforming growth factor-α in human esophageal carcinoma. *Cancer* **71:** 2902–2909

Imazeki F, Omata M, Nose H, Ohto M and Isono K (1992) p53 gene mutations in gastric and esophageal cancers. *Gastroenterology* **103:** 892–896

Jakl RJ, Miholic J, Koller R, Markis E and Wolner E (1995) Prognostic factors in adenocarcinoma of the cardia. *Am J Surg* **169:** 316–319

Kajiyama Y, Tsurumaru M, Udagawa H, Tsutsumi K, Kinoshita Y, Ueno M and Akiyama H (1997) Prognostic factors in adenocarcinoma of the gastric cardia: pathologic stage analysis and multivariate regression analysis. *J Clin Onc* **15:** 2015–2021

© 2000 Cancer Research Campaign 

MacFarlane SD, Hill LD, Jolly PE, Kozarek RA and Anderson RP (1988) Improved results of surgical treatment for oesophageal and gastroesophageal carcinoma after preoperative combined chemotherapy and radiation. *J Thorac Cardiovasc Surg* **95:** 415–422

Malden LT, Novak U and Burgess AW (1989) Expression of transforming growth factor alpha messenger mRNA in the normal and neoplastic gastro-intestinal tract. *Int J Cancer* **43:** 380–384

Martin HM, Filipe MI and Morris RW (1992) p53 expression and prognosis in gastric carcinoma. *Int J Cancer* **50:** 859–862

Moskaluk A, Heitmiller R, Zahurak M, Schwab D, Sidransky D and Hamilton SR (1996) p53 and p21waf1/cip1 gene products in Barrett's esophagus and adenocarcinoma of the esophagus and the esophagogastric junction. *Hum Pathol* **27:** 1211–1220

Naito Y, Saito K, Shiba K, Ohuchi A, Saigenji K, Nagura H and Ohtani H (1998) CD8+ T cells infiltrated within cancer cell nests as a prognostic factor in human colorectal cancer. *Cancer Res* **58:** 3491–3494

Nasim MM, Thomas DM, Alison MR and Filipe MI (1992) Transforming growth factor α expression in normal gastric mucosa, intestinal metaplasia, dysplasia and gastric carcinoma–an immunohistochemical study. *Histopathology* **20:** 339–343

Ohno S, Tomisaki S and Oiwa H (1995) Clinicopathologic characteristics and outcome of adenocarcinoma of the human gastric cardia in comparison with carcinoma of other regions of the stomach. *J Am Coll Surg* **180:** 577–582

Patel DD, Bhatavdekar JM, Chikhlikar PR, Patel YV, Shah NG, Ghosh N, Suthar TP and Balar DB (1997) Clinical significance of p53, nm23, and bcl-2 in T3-4N1M0 oesophageal carcinoma. An immunohistochemical approach. *J Surg Oncol* **65:** 111–116

Paterson IM, Easton DF, Corbishely CM and Gazet JC (1987) Changing distribution of adenocarcinoma of the stomach. *Br J Surg* **74:** 481–482

Pera M, Cameron AJ, Trastek VF, Carpenter HA and Zinmeister AR (1993) Increasing incidence of adenocarcinoma of the esophagus and esophagogastric junction. *Gastroenterology* **104:** 510–513

Powell J and McConkey CC (1992) The rising trend in oesophageal adenocarcinoma and gastric cardia. *Eur J Cancer Prev* **1:** 265–269

Ropponen KM, Eskelinen MJ, Lipponen PK, Alhava E and Kosma VM (1997) Prognostic value of tumour-infiltrating lymphocytes (TILs) in colorectal cancer. *J Pathol* **182:** 318–324

Sauter ED, Coia LR, Eisenberg BL and Hanks GE (1995) Transforming growth factor-α expression as a potential survival prognosticator in patients with esophageal adenocarcinoma receiving high-dose radiation and chemotherapy. *Int J Radiat Oncol Biol Phys* **31:** 567–569

Schreiber AB, Winkler ME and Derynk R (1986) Transforming growth factor-α: a more potent angiogenic mediator than epidermal growth factor. *Science* **232:** 1250–1253

Sharpe DAC and Moghissi K (1996) Resectional surgery in carcinoma of the oesophagus and cardia: what influences long-term survival? *Eur J Cardio-thorac Surg* **10:** 359–364

Siewert JR and Stein HJ (1996) Carcinoma of the cardia: carcinoma of the gastroesophageal junction – classification, pathology and extent of resection. *Dis Esophagus* **9:** 173–182

Sons HU and Borchard F (1986) Cancer of the distal esophagus and cardia: incidence, tumorous infiltration, and metastatic spread. *Ann Surg* **203:** 188–195

Stemmermann G, Heffelfinger SC, Noffsinger A, Hui YZ, Miller MA and Fenoglio-Preiser C (1994) The molecular biology of esophageal and gastric cancer and their precursors: oncogenes, tumour suppressor genes, and growth factors. *Hum Pathol* **25:** 968–981

Steup WH, De Leyn P, Deneffe G, Van Raemdonck D, Coosemans W and Lerut T (1996) Tumours of the esophagogastric junction. Long-term survival in relation to the pattern of lymph node metastasis and a critical analysis of the accuracy or inaccuracy of pTNM classification. *J Thorac Cardiovasc Surg* **111:** 85–95

Wang HH, Antonioli DA and Goldman H (1986) Comparative features of esophageal and gastric adenocarcinomas: recent changes in type and frequency. *Hum Pathol* **17:** 482–487

Wang LD, Shi ST, Zhou Q, Goldstein S, Hong JY, Shao P, Qiu SL and Yang CS (1994) Changes in p53 and cyclin D1 protein levels and cell proliferation in different stages of human esophageal and gastric-cardia carcinogenesis. *Int J Cancer* **15:** 514–519

Wang L-S, Wu C-W, Hsieh M-J, Fahn H-J, Huang M-H and Chien K-Y (1993) Lymph node metastasis in patients with adenocarcinoma of gastric cardia. *Cancer* **71:** 1948–1953

Webb JN and Busuttil A (1978) Adenocarcinoma of the oesophagus and of the oesophagogastric junction. *Br J Surg* **65:** 475–480

Wolfe WG, Vaughan AI, Siegler HF, Hawthorne JW, Leopold KA and Duhay-Longsod FG (1988) Survival of patients with carcinoma of the oesophagus treated with combined-modality therapy. *J Thorac Cardiovasc Surg* **105:** 749–756

Wu TT, Watanabe T, Heitmiller R, Zahurak M, Forastiere AA and Hamilton SR (1998) Genetic alterations in Barrett esophagus and adenocarcinomas of the esophagus and esophagogastric junction region. *Am J Pathol* **153:** 287–294

Yonemura Y, Takamura H, Ninomiya I, Fushida S, Tsugawa K, Kaji M, Nakai Y, Ohoyama S, Yawaguchi A and Miyazaki I (1992) Interrelationship between transforming growth factor-α and epidermal growth factor receptor in advanced gastric cancer. *Oncology* **49:** 157–161

Younes M, Fernandez L and Lechago J (1996) Transforming growth factor alpha (TGF-α) expression in biopsies of colorectal carcinoma is a significant prognostic indicator. *Anticancer Res* **16:** 1999–2004

 *© 2000 Cancer Research Campaign*
